# Role of Ultrasound-Measured Diaphragmatic Excursion and Thickness in Predicting Extubation Outcomes in Perforation Peritonitis Patients: A Comparative Observational Study

**DOI:** 10.7759/cureus.93570

**Published:** 2025-09-30

**Authors:** Jayaram Ilangovan, Stalin Vinayagam, Sandeep Kumar Mishra, Senthilnathan Muthapillai

**Affiliations:** 1 Anesthesiology, Mahatma Gandhi Medical College and Research Institute, Puducherry, IND; 2 Anesthesiology and Critical Care, Jawaharlal Institute of Postgraduate Medical Education and Research, Puducherry, IND

**Keywords:** diaphragm, extubation, laparotomy, peritonitis, ultrasonography

## Abstract

Background

Perforation peritonitis, a common surgical emergency, is associated with postoperative respiratory complications. This study aimed to measure diaphragmatic excursion and thickness using preoperative ultrasound in patients undergoing laparotomy for perforation peritonitis, thereby predicting extubation outcomes at the end of the surgery.

Methodology

A total of 80 patients aged 18-60 years, belonging to the American Society of Anesthesiologists physical class I-III, scheduled to undergo laparotomy under general anesthesia were included in this study. The study sample was divided into the following two groups (40 in each group): Group P, perforation peritonitis patients undergoing emergency laparotomy, and Group C, patients undergoing elective laparotomy. Preoperatively, diaphragm excursion on deep inspiration and diaphragmatic thickness at end-inspiration and end-expiration were measured using ultrasound in both groups. Respiratory rate, arterial blood gas analysis, abdominal girth, duration of surgery, extubation outcome, and duration of mechanical ventilation were recorded and compared between the groups.

Results

Diaphragm excursion was significantly reduced in Group P (1.62 cm) compared to Group C (3.3 cm) (p < 0.001). Diaphragm thickness at end-inspiration and end-expiration was significantly reduced in Group P compared to Group C (p = 0.008). In Group P, diaphragm thickness at end-inspiration and end-expiration was significantly higher in those who were successfully extubated. From the receiver operating characteristic curve analysis, a diaphragmatic thickness cut-off value of 0.15 cm at end-inspiration and 0.14 cm at end-expiration predicted successful extubation in Group P.

Conclusions

In this study, diaphragmatic excursion and thickness were significantly reduced in patients with perforation peritonitis. Hence, diaphragmatic thickness can be a useful predictor of extubation outcomes at the end of the surgery.

## Introduction

Perforation peritonitis is one of the most common surgical emergencies in India, and these patients develop respiratory complications after emergency laparotomy [1]. Preoperative atelectasis is the main cause that leads to postoperative mechanical ventilation and intensive care unit admissions [2]. Assessment of diaphragmatic excursion and thickness in these patients in the postoperative period leads to a significant decrease in diaphragmatic measurements. This reduction may be responsible for atelectasis, reduced vital capacity, and hypoxemia in the postoperative period. Preoperative identification of diaphragm dysfunction in these patients can help predict postoperative respiratory complications. There are many tools to assess diaphragmatic excursion and thickness. However, they have their limitations, such as the risk of ionizing radiation (fluoroscopy, CT) or their complex and highly specialized nature requiring a skilled operator (trans-diaphragmatic pressure measurement, diaphragmatic electromyography, phrenic nerve stimulation, MRI) [3].

Ultrasound is a novel and non-invasive method for diagnosing diaphragmatic dysfunction and has proven to be an accurate, safe, easy-to-use bedside modality, overcoming many of the limitations of other imaging techniques. Ultrasonography can assess the characteristics of the diaphragmatic movement and changes in diaphragmatic thickness during respiration, which can provide valuable information for assessing patients with diaphragmatic dysfunction.

The primary objective of this study was to compare diaphragmatic excursion and thickness in patients with perforation peritonitis and those undergoing elective laparotomy using preoperative ultrasound. The secondary objective was to correlate the ultrasonography measurements with extubation outcomes in patients with perforation peritonitis.

## Materials and methods

This comparative observational study was conducted between February 2019 and March 2021 after obtaining approval from the Institute Ethics Committee, Jawaharlal Institute of Postgraduate Medical Education and Research (approval number: JIP/IEC/2018/468). This study was conducted in accordance with the Declaration of Helsinki. Written informed consent was obtained from all patients who participated in the study. Perforation peritonitis patients scheduled for emergency laparotomy and patients posted for elective laparotomy between the age group of 18-60 years and American Society of Anesthesiologists (ASA) grades I-III were enrolled in this study after fulfilling eligibility criteria. Patients who were intubated and those with pneumothorax, pneumomediastinum, and pre-existing pulmonary or neuromuscular disease were excluded from the study. The study population was divided into the following two groups: Group P and Group C. Group P included patients with perforation peritonitis scheduled for emergency laparotomy under general anesthesia. Group C included patients scheduled for elective laparotomy under general anesthesia.

**Figure 1:**
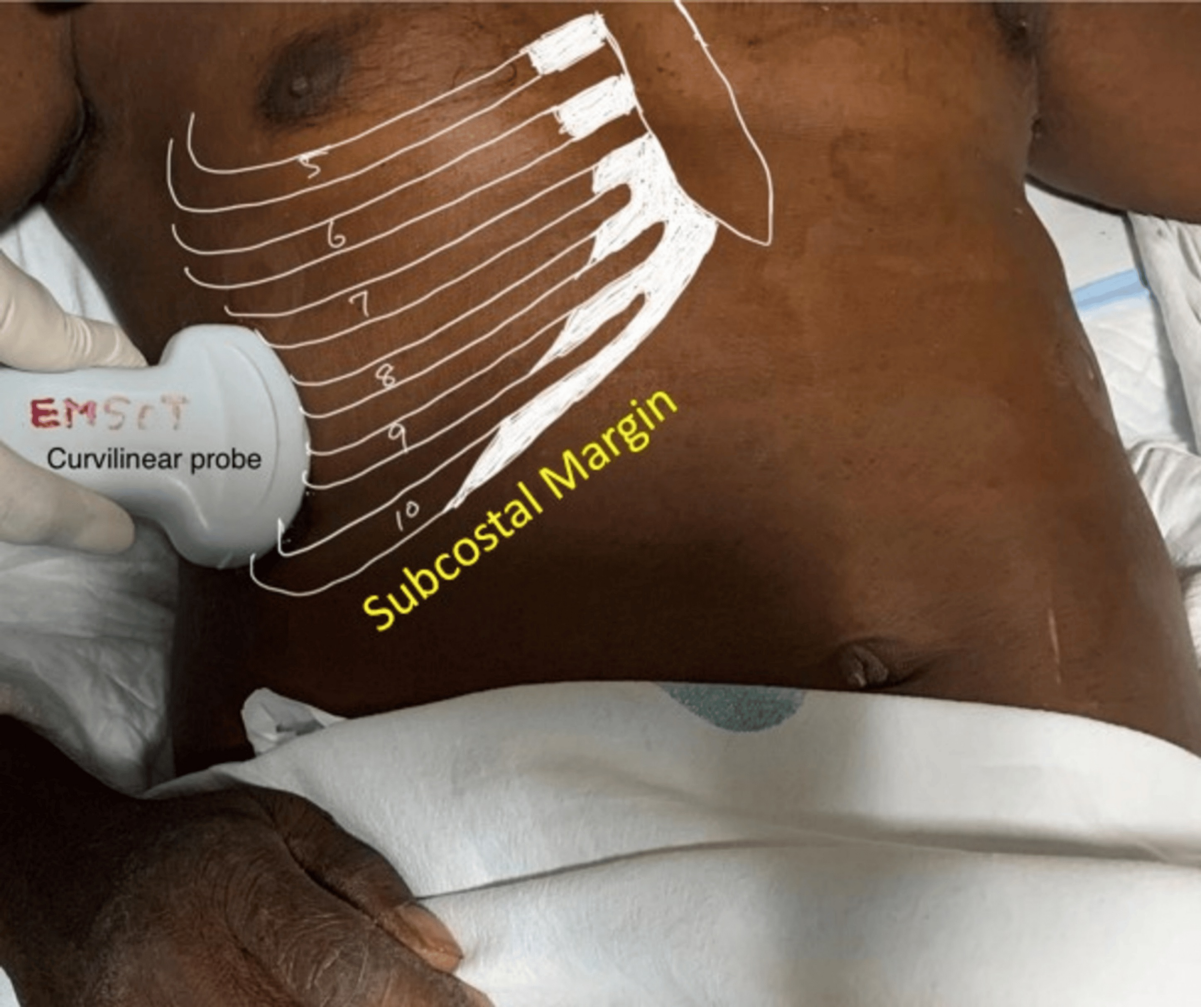
Probe position for visualizing right diaphragmatic excursion with the curvilinear probe.

**Figure 2:**
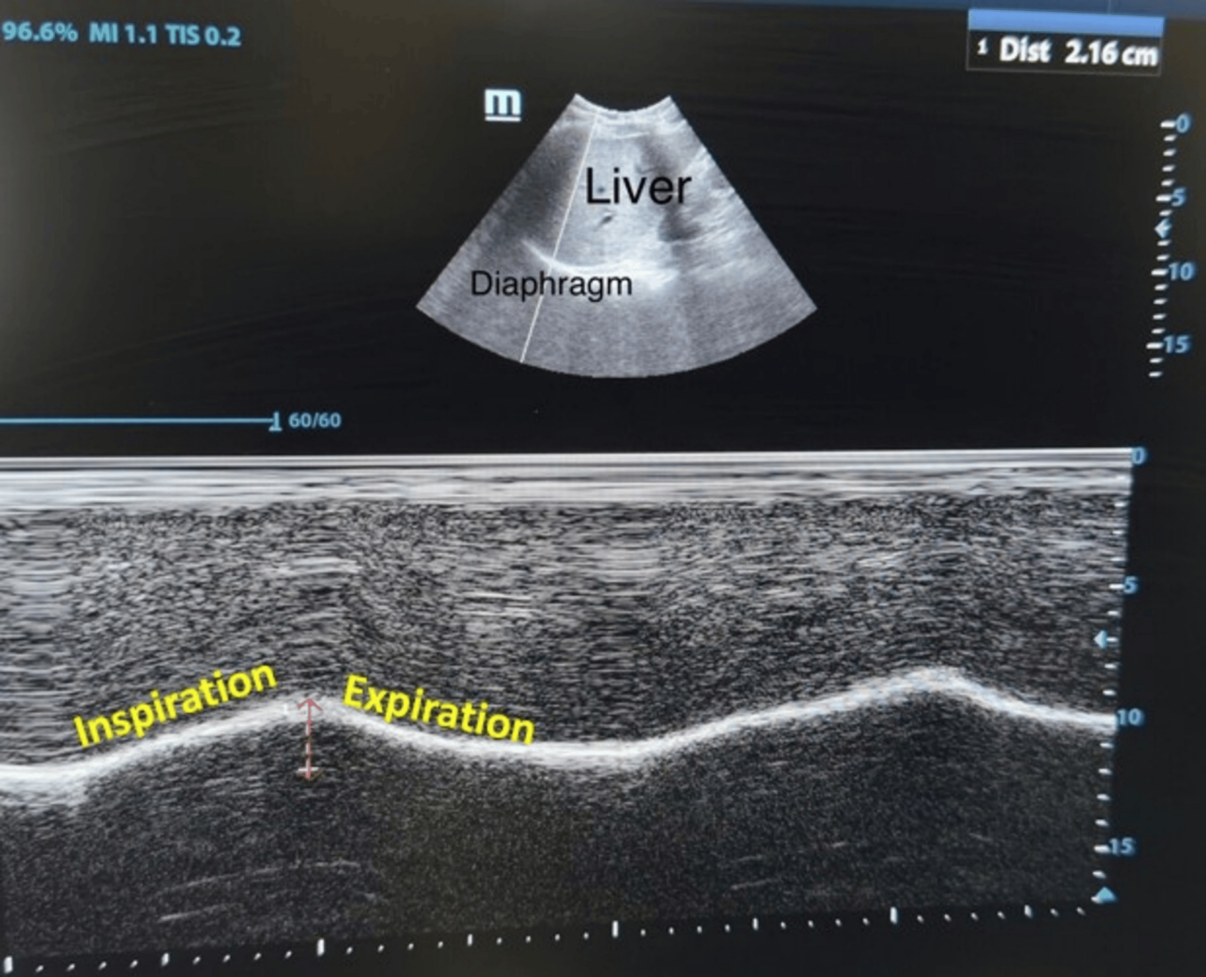
Right diaphragmatic excursion measured using M-mode sonography with the curvilinear probe.

**Figure 3:**
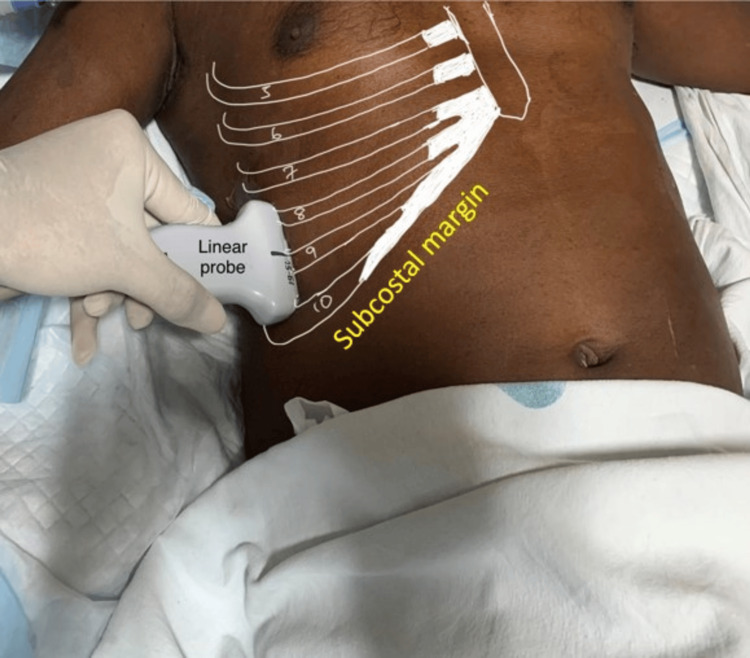
Probe position for visualizing right diaphragmatic thickness with the linear probe.

**Figure 4:**
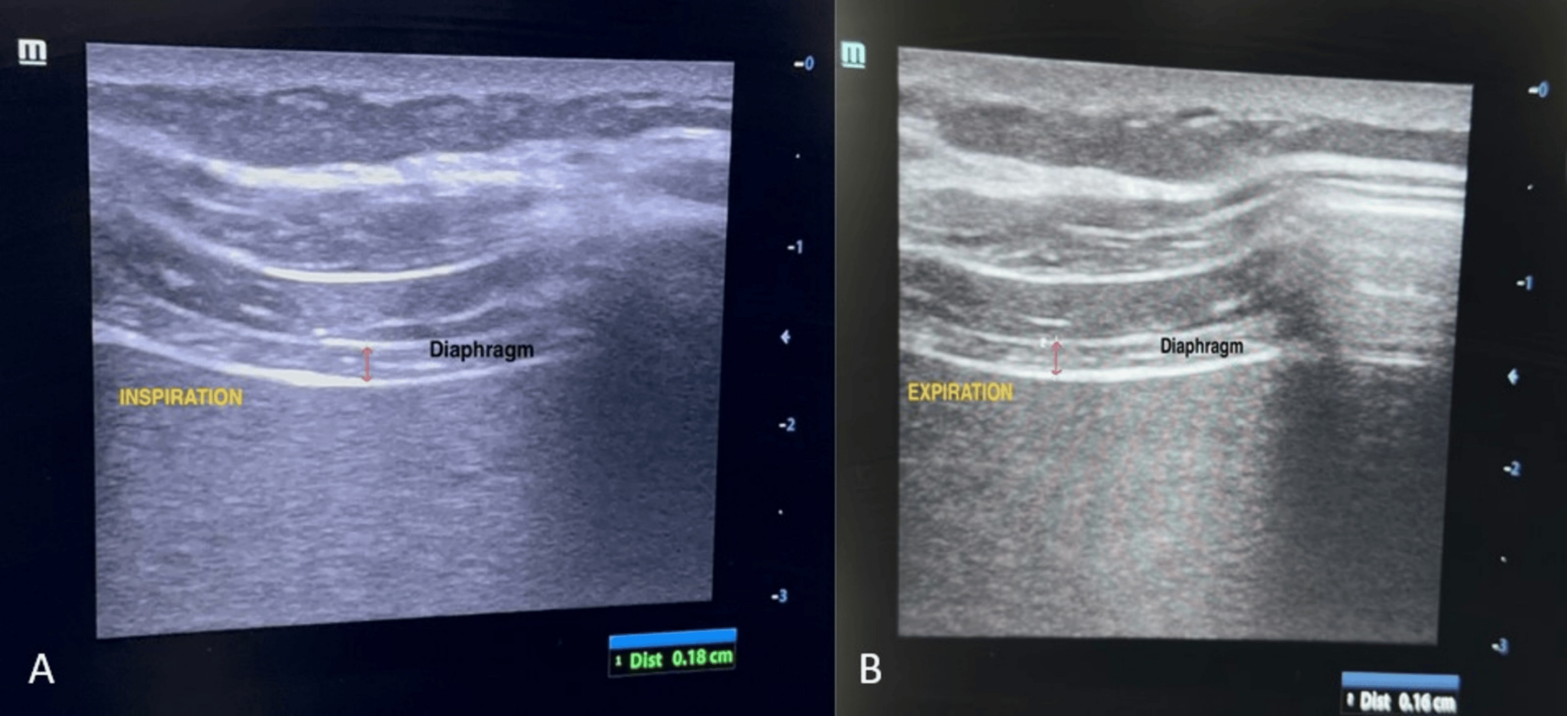
Right diaphragm thickness measured using B-mode sonography with the linear probe at end-inspiration (A) and end-expiration (B).

The attending anesthesiologist obtained a history of the duration of the illness and recorded demographic data. All standard monitors were attached in the operating room, and baseline parameters were recorded. In the supine position, a trained anesthesiologist performed an ultrasound examination and visualized the diaphragm using a convex phased array probe (bandwidth 2-5 MHz) of a portable ultrasound machine (Fujifilm SonoSite Inc., Bothell, WA, USA). With B-mode set as the default mode on the device screen, the probe was placed longitudinally on the right anterior axillary line at the level of the eighth to tenth intercostal space (Figure 1) to achieve the best view of the right hemidiaphragm. The right hemidiaphragm was selected as the liver serves as an acoustic window. At the zone of apposition, switching to M-mode, the patient was asked to take a deep inspiration, and the measurement was obtained. Inspiration was identified on the sonographic tracing as an upward flexion, and expiration was identified as a downward flexion. The diaphragmatic excursion was measured by the vertical distance from the baseline to the point of maximum height of inspiration (Figure 2). With the device in B-mode and using a linear array probe at the same point (Figure 3), the right hemidiaphragm was identified, and the vertical distance between the pleural and peritoneal lines was noted (Figures 4A, 4B). This diaphragm thickness was measured at end-inspiration and end-expiration. Diaphragmatic thickness fraction (dtf) was calculated using the following formula: dtf = thickness at end-inspiration - thickness at end-expiration/ thickness at end-expiration × 100.

Parameters such as respiratory rate, arterial blood gas analysis (ABG), and abdominal girth were also noted before induction. General anesthesia was induced as per departmental protocol. At the end of the surgery, the duration of surgery and the extubation outcome were recorded. The extubation outcome mentioned whether the patient would be extubated at the end of the surgery or not. If not extubated, the duration of mechanical ventilation was noted. The preoperative ultrasound measurements were purely observational and were not used to decide extubation outcomes. Standardized criteria, such as clinical assessment of recovery, ABG parameters, and hemodynamic status, were used to decide the extubation outcome.

Statistical analysis

The sample size was estimated using the statistical formula to compare two independent means. With reference to Ali et al., the difference in mean diaphragmatic excursion between the groups was 0.9 cm with an SD of 1.44 cm [4]. For an alpha error of 5% and a power of 80%, the estimated sample size was 40 in each group. Statistical analysis was performed using SPSS version 21.0 (IBM Corp., Armonk, NY, USA). Categorical variables such as sex, extubation outcome, and mortality rate were expressed as frequency and percentage. Continuous variables such as age, weight, height, duration of illness, body mass index (BMI), ABG values, abdominal girth, duration of surgery, diaphragm excursion, and diaphragm thickness at end-expiration, and diaphragmatic thickness at end-inspiration were expressed as mean with SD. Distribution of respiratory rate, diaphragmatic thickness fraction, and duration of mechanical ventilation was expressed in terms of the median with interquartile range. The comparison of frequency and percentage was performed using the chi-square test. Comparison of the mean was made with the independent t-test, and median with the Mann-Whitney U-test. The correlation between the continuous variables was expressed by the Karl Pearson correlation coefficient or the Spearman rank correlation coefficient. A p-value <0.05 was considered statistically significant.

## Results

A total of 80 patients were recruited for the study. The demographic data were comparable between the groups (Table 1). Group P had a significantly higher median respiratory rate and mean abdominal girth (p < 0.001). Arterial blood gas showed significantly lower PCO_2_ and HCO_3_ and higher lactate in Group P compared to Group C (p < 0.001) (Table 2).

**Table 1:** Comparison of demographic parameters between Group P and Group C. Age, height, weight, and BMI are expressed as mean ± SD. Gender and ASA category are expressed as frequency and percentage (%). ASA = American Society of Anesthesiologists; BMI = body mass index

Parameter		Group P	Group C
Age		48 ± 11	45 ± 11
Height (cm)		165 ± 7	166 ± 6.4
Weight (kg)		62 ± 13.4	62.9 ± 12.1
BMI (kg/m^2^)		22.4 ± 4.2	22.7 ± 4.7
Gender	Male	30/40 (75%)	32/40 (80%)
	Female	10/40 (25%)	8/40 (20%)
ASA category	I	20/40 (50%)	22/40 (55%)
	II	15/40 (37.5%)	17/40 (42.5%)
	III	5/40 (12.5%)	1/40 (2.5%)

**Table 2:** Comparison of baseline parameters between Group P and Group C. Respiratory rate is expressed as median (interquartile range). Arterial blood gas values, abdominal girth, and duration of surgery are expressed as mean (SD). P-values <0.05 are statistically significant.

Variables	Group P (n = 40)	Group C (n = 40)	P-value
Respiratory rate (per minute)	24 (20–29)	16 (16–18)	<0.001
pH	7.39 (0.08)	7.42 (0.03)	0.075
pCO_2_ (mmHg)	30.1 (6)	33.9 (2.6)	<0.001
pO_2_ (mmHg)	91.8 (13.3)	91.9 (7.8)	0.944
HCO_3_ (mEq/L)	18.9 (5.2)	23.6 (2.1)	<0.001
Lactate (mmol/L)	2.4 (1.6)	1.2 (0.5)	<0.001
Abdominal girth (cm)	82 (13)	67 (9.5)	<0.001
Duration of surgery (hours)	3.5 (1.9)	4.4 (1.8)	0.026

**Figure 5:**
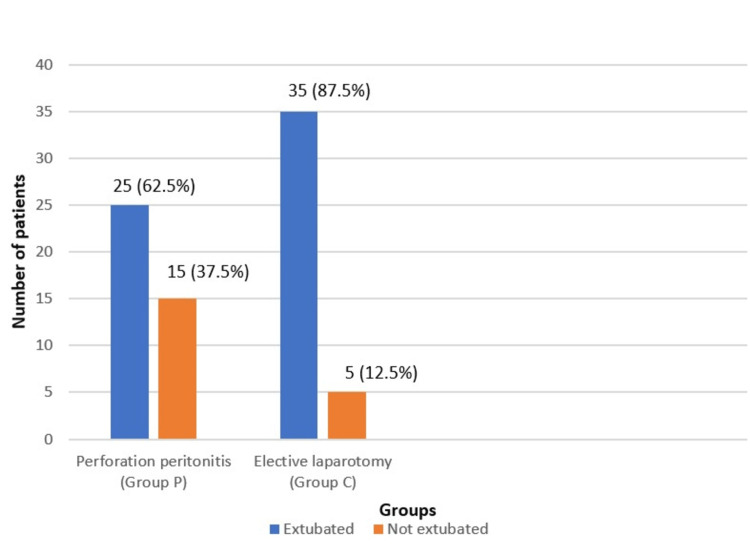
Comparison of extubation outcomes between Group P and Group C.

The mean diaphragmatic excursion during deep inspiration was significantly lower in Group P (1.62 cm) compared to Group C (3.3 cm) (p < 0.001). Similarly, the mean diaphragmatic thickness at the end-inspiration and end-expiration were also significantly lower in Group P. However, there was no significant difference in the median diaphragmatic thickness fraction between the two groups (p = 0.630) (Table 3). Successful extubation after surgery was also significantly lower in Group P (p = 0.01) (Figure 5).

**Table 3:** Comparison of diaphragmatic parameters between Group P and Group C. Diaphragmatic excursion and thickness are expressed as mean (SD). Diaphragmatic thickness fraction is expressed as median (interquartile range). P-values <0.05 are statistically significant.

Variables	Group P (n = 40)	Group C (n = 40)	P-value
Diaphragm excursion (cm)	1.62 (0.82)	3.3 (1.5)	<0.001
Diaphragm thickness inspiration (cm)	0.179 (0.036)	0.203 (0.039)	0.008
Diaphragm thickness expiration (cm)	0.158 (0.033)	0.177 (0.034)	0.013
Diaphragm thickness fraction (%)	10.5 (7.7–15.5)	11.4 (6.7–19.8)	0.630

**Figure 6:**
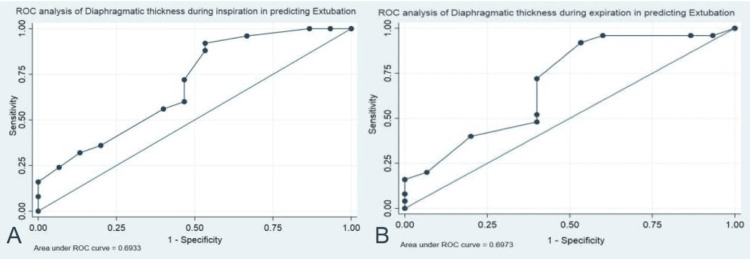
Receiver operating characteristic curve of diaphragmatic thickness at end-inspiration (A) and end-expiration (B) predicting extubation outcomes in the perforation peritonitis group.

The diaphragmatic excursion and thickness were correlated with extubation outcome in the Group P (Table 4). A significant positive correlation was noted between diaphragmatic thickness and successful extubation outcome (Spearman correlation = 0.348, p = 0.002). Subgroup analysis of Group P patients found that diaphragm thickness at end-inspiration and end-expiration was significantly higher in extubated patients than in non-extubated patients (Table 5). Receiver operating characteristic (ROC) curve analysis was done for diaphragmatic thickness at end-inspiration to predict extubation in patients with perforation peritonitis. A cut-off value of >0.15 cm of diaphragmatic thickness at end-inspiration has a sensitivity of 92% and specificity of 46.7% to predict successful extubation in perforation peritonitis patients (Figure 6A). ROC curve analysis was also performed for diaphragmatic thickness at end-expiration to predict extubation in patients with perforation peritonitis. A cut-off value of >0.14 cm of diaphragmatic thickness at end-expiration has a sensitivity of 92% and specificity of 46.7% to predict successful extubation in patients with perforation peritonitis (Figure 6B).

**Table 4:** Correlation between diaphragmatic measurements and extubation outcomes in the perforation peritonitis group. P-values <0.05 are statistically significant.

Parameters	Spearman correlation coefficient	P-value
Diaphragm excursion and successful extubation outcome	0.221	0.049
Diaphragm thickness and successful extubation outcome	0.348	0.002

**Table 5:** Comparison of variables between extubated and not extubated patients in the perforation peritonitis group, Diaphragmatic excursion and thickness are expressed in mean (SD). Diaphragmatic thickness fraction is expressed in median (interquartile range). P-values <0.05 are statistically significant.

Parameters	Extubated patients (n = 25)	Not extubated patients (n = 15)	P-value
Diaphragm excursion (cm)	1.75 (0.86)	1.41 (0.72)	0.211
Diaphragm thickness inspiration (cm)	0.189 (0.034)	0.163 (0.036)	0.024
Diaphragm thickness expiration (cm)	0.166 (0.032)	0.145 (0.031)	0.042
Diaphragm thickness fraction (%)	11.8 (7.7–16.7)	9.09 (7.7–14.3)	0.370

**Figure 7:**
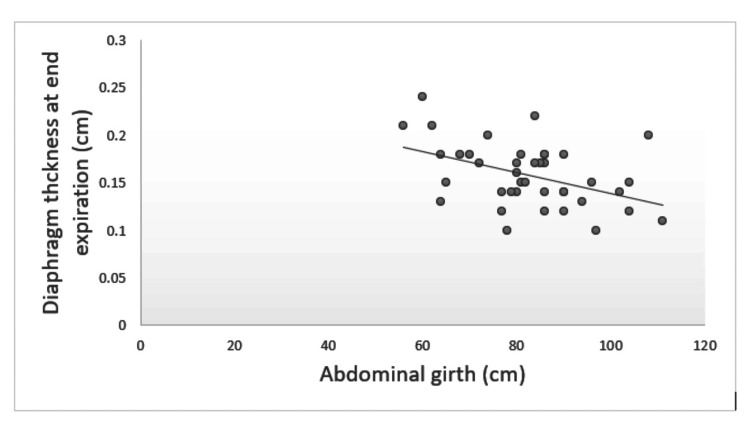
Association between abdominal circumference and diaphragmatic thickness at end-expiration in the perforation peritonitis group. Correlation between diaphragm thickness at end expiration and abdominal girth was done with Pearson correlation coefficient with a value of -0.447 (p = 0.004).

There was a significant negative correlation between abdominal girth and diaphragmatic thickness at the end-expiration (Figure 7). In patients who were not extubated, there was a significant correlation between diaphragmatic thickness at the end-inspiration and duration of mechanical ventilation (p = 0.004).

## Discussion

This study found a significant reduction in diaphragmatic excursion and diaphragmatic thickness in patients with perforation peritonitis. We also found that a diaphragmatic thickness of >0.15 cm at end-inspiration can predict successful extubation in patients with perforation peritonitis. There was also a significant negative correlation between abdominal girth and diaphragmatic thickness at end-expiration.

Abdominal distension in patients with perforation peritonitis can affect the mobility of the diaphragm, resulting in diaphragmatic dysfunction [1]. Increased intra-abdominal pressure increases chest wall elastance (or decreases compliance) and promotes cephalad shift of the diaphragm, with a consequent reduction in lung volumes and atelectasis formation [5]. Studies with magnetometers show a decrease in the ratio of changes in abdominal to rib cage diameters, with a shift from predominantly abdominal to thoracic breathing in the postoperative period after open cholecystectomy, which indicates decreased diaphragmatic excursion and thickness. Difficulty in weaning from mechanical ventilation and the development of postoperative respiratory complications are common outcomes in patients with such diaphragmatic dysfunction.

Our study has found that diaphragmatic excursion during deep inspiration was significantly reduced in patients with perforation peritonitis, with a mean value of 1.75 cm in extubated patients. Similar results were found by Saravanan et al., where a diaphragmatic excursion value of >1.21 cm had a sensitivity of 94% and specificity of 71% for predicting successful weaning in intensive care unit patients on mechanical ventilation [6]. In contrast to our finding, many studies have found a mean diaphragmatic excursion ranging from 2.7 cm to 3.75 cm in successfully extubated patients [7-9]. This difference could be because of the study population included in these studies, which was chronically ventilated patients in the intensive care unit who had successful weaning, compared to non-intubated perforation peritonitis patients with diaphragmatic dysfunction in our study.

Diaphragmatic thickness at end-inspiration in perforation peritonitis patients was found to be 0.179 cm in our study. Similar to our study, Chen et al. measured the diaphragmatic thickness at end-expiration in sepsis patients who were not on mechanical ventilation and found it to be 0.20 cm [7]. Studies have also reported a mean diaphragmatic thickness at end-inspiration of 0.20 cm in intubated critically ill patients [10-12]. In our study, diaphragmatic thickness at the end-inspiration value of > 0.15 cm had a sensitivity of 92% and specificity of 46.7%, and diaphragmatic thickness at the end-expiration value of > 0.14 cm had a sensitivity of 92% and specificity of 46.7% in predicting successful extubation in perforation peritonitis patients. Similar to our findings, many studies have also shown that a diaphragmatic thickness at end-inspiration of >0.20 cm had a good predictive value for successful extubation in chronically intubated intensive care patients [4,9].

In the study by Blumhof et al., diaphragmatic thickness change (Δtdi) of >20% was a robust predictor of extubation success [13]. This contradicts our study, where the median diaphragmatic thickness fraction in extubated patients with perforation peritonitis was 11.8%. This could be due to the reduced diaphragm thickness because of abdominal distension and chronic respiratory distress in patients with perforation peritonitis. We also found that abdominal girth negatively correlated with diaphragm thickness at end-inspiration and end-expiration, which was statistically significant. This correlation implies that increased abdominal girth causes a decrease in diaphragmatic thickness. In our study, the diaphragm thickness at end-inspiration and end-expiration was also positively correlated with the duration of mechanical ventilation. This correlation implies that we expect an increased duration of mechanical ventilation in patients with increased diaphragm thickness values and vice versa. This false correlation can be attributed to confounding factors such as the severity of hemodynamic instability and severe sepsis with metabolic acidosis, which increases the duration of mechanical ventilation even in patients with higher diaphragm thickness values.

Though our study is the first attempt to assess diaphragmatic excursion and thickness with ultrasonography in perforation peritonitis patients, it is not without limitations. First, the diaphragm thickness value to predict extubation had a low specificity of 46.7% making it a weak standalone predictor. Second, we did not take into account pain while assessing diaphragm excursion, which could be a confounding factor limiting diaphragm mobility in patients with perforation peritonitis. Third, intra-abdominal pressure was not objectively measured in our study. As high intra-abdominal pressure could severely hamper diaphragmatic excursion, future research should explore measuring intra-abdominal pressure to evaluate its impact on diaphragmatic measurements. Fourth, comparing emergency laparotomy patients with elective laparotomy patients introduces several confounding variables, such as pain and abdominal distension. We preferred elective laparotomy patients as comparators as these patient populations have closer similarities with perforation peritonitis patients compared to healthy volunteers. Fifth, an ultrasound assessment of the diaphragm was performed only once in the preoperative holding area. Factors such as patient cooperation and interobserver variability were not assessed in our study, which could have influenced our measurements. Sixth, the preoperative pulmonary function test was not performed, which could have helped correlate with diaphragmatic measurements. Seventh, neuromuscular monitoring was not used to assess the level of blockade before extubation, as objective confirmation of complete recovery from neuromuscular blockade is crucial when evaluating extubation outcomes, and the absence of such monitoring may have influenced results attributed to diaphragmatic excursion and thickness. Finally, the anesthesiologist was not blinded, and there was a risk of observation bias.

Future studies with larger sample sizes are required, incorporating additional tests mentioned above, correlating with postoperative pulmonary complications, and weaning from ventilation. There should be appropriate control group selection and elimination of confounding variables to translate the findings into clinical practice. Population groups, such as the elderly (more than 60 years), obese, and patients with chronic pulmonary disease, should be included in future studies to enhance generalizability.

## Conclusions

We conclude that the diaphragmatic excursion and thickness were significantly reduced in patients with perforation peritonitis. Moreover, diaphragmatic thickness at end-inspiration and end-expiration can be a useful predictor of extubation outcomes at the end of the surgery.
